# Re-engineering the disordered mind: clinical experimentation, dynamical systems, and AI for personalized psychiatry

**DOI:** 10.1038/s41386-025-02303-z

**Published:** 2025-12-17

**Authors:** Mina Kheirkhah, Bita Shariatpanahi, Tim Hahn, Vineet Tiruvadi, Georgia Koppe, Erfan Nozari, Stefan G. Hofmann, Hamidreza Jamalabadi

**Affiliations:** 1 Department of Psychology, Marburg University, Marburg, Germany; 2 Department of Psychiatry and Psychotherapy, Jena University Hospital, Jena, Germany; 3 Department of Psychiatry and Psychotherapy, Marburg University, Marburg, Germany; 4 Institute for Translational Psychiatry, University of Münster, Münster, Germany; 5 Department of Neurology, Harvard Medical School, Boston, MA USA; 6 Interdisciplinary Center for Scientific Computing, Heidelberg University, Heidelberg, Germany; 7 Clinic for Psychiatry and Psychotherapy, Central Institute of Mental Health, Medical Faculty, Heidelberg University, Mannheim, Germany; 8 Hector Institute for AI in Psychiatry, Central Institute of Mental Health, Medical Faculty, Mannheim, Germany; 9 Department of Mechanical Engineering, University of California, Riverside, CA USA; 10 Department of Electrical and Computer Engineering, University of California, Riverside, CA USA; 11 Department of Bioengineering, University of California, Riverside, CA USA; 12 Neuroscience Graduate Program, University of California, Riverside, CA USA; 13 Center for Mind, Brain, and Behavior (CMBB), Marburg University, Marburg, Germany; 14 Faculty of Medicine, University of British Columbia, Vancouver, BC Canada

**Keywords:** Neuroscience, Medical research, Biomarkers, Psychology

## Abstract

This perspective proposes a neuropsychiatric model of psychological and psychiatric interventions by reframing treatment as a control engineering problem grounded in dynamical systems theory and artificial intelligence (AI). We argue that psychopathology arises from distortions in the geometry of underlying neurobehavioral low-dimensional cognitive–affective manifolds rather than from isolated biological dysfunctions, and we use a formal dynamical framework to show how clinical interventions can be modeled as control inputs that reshape the manifold itself to restore healthy dynamics. To operationalize this approach clinically, we propose a closed-loop, N-of-1 experimental paradigm in which dense longitudinal measurements and strategically designed perturbations are used to train individualized AI surrogate models of a person’s manifold. This model supports the simulation of counterfactual interventions and guide the design of optimized, personalized treatments. Active perturbation reduces required sample size dramatically, enabling precise modeling from limited but richly sampled individual data. This engineering-inspired framework reconceptualizes clinical improvement as the restoration of regulatory capacity and resilient trajectories rather than the mere reduction of symptom counts. By integrating dynamical systems theory, AI-based surrogate modeling, and adaptive clinical experimentation, we outline a principled pathway toward personalized neuropsychiatry based on dynamical systems theory and AI.

Despite decades of research, progress in neuropsychiatric treatment has plateaued, and efforts to identify reliable biomarkers have often produced inconsistent or non-replicable results [1, 2]. This persistent challenge suggests that current conceptual frameworks may be limited, calling for a fundamental rethinking of how we understand and intervene in mental disorders.

Evidence from neuroimaging and AI now points toward a promising alternative: both neural activity and behavior evolve on reproducible low-dimensional manifolds, shaped by interactions across brain, body, and environment [3]. These findings motivate a shift from symptom- or network-based models toward a geometry-based framework for psychiatry.

In this framework, mental states are conceived not as static entities but as dynamic trajectories on smooth, embedded manifolds within high-dimensional state space. This marks a key departure from mainstream neuropsychiatric models: we posit that mental states themselves—such as the experience of mental well-being—are time-evolving trajectories in a dynamical systems framework (see Supplementary Table 1 for definitions of related terminology and their relation to neuropsychiatry). For example, in the simplified linear case (Eqs. 4, 5), mental well-being may correspond to an eigenmode with a characteristic time constant governed by the system’s eigenvalue. The geometry of these manifolds, defined by their topology and curvature, thus shapes how cognitive and affective processes evolve over time (Fig. 1A). Psychopathology, in turn, may not be reducible to localized deficits or isolated dysfunctions; rather, it emerges from maladaptive manifold landscapes. We, therefore, use the term “features of psychopathology” instead of symptoms; a second critical distinction. Unlike the symptom concept, which presupposes an underlying latent disease entity, our manifold perspective does not assume such a hidden substrate. Instead, it treats psychopathology as observable trajectories in cognitive–affective and behavioral state spaces. Features, therefore, refer to measurable expressions of psychopathology without committing to a disease-centered ontology. For example, depressive states may correspond to multiple overlapping basins of attraction whose boundaries do not map neatly onto individual features (and by extension onto symptoms). This perspective aligns with transdiagnostic initiatives such as Research Domain Criteria (RDoC [4]), which emphasize mechanisms and processes over categorical checklists.

**Fig. 1:**
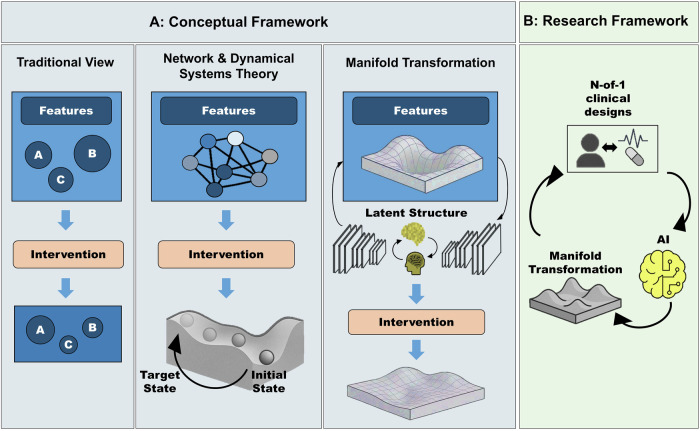
From features to manifolds: a dynamical framework for personalized psychiatry. **A** Conceptual frameworks for understanding and treating mental disorders. Traditional View (left): Disorders are described as collections of discrete features of psychopathology (which are traditionally conceptualized as symptoms), with interventions targeting each in isolation. Network & Dynamical Systems (center): Features of psychopathology emerge from interacting networks; interventions aim to shift trajectories within an underlying landscape. Manifold Transformation (right): Features of psychopathology reflect trajectories on low-dimensional manifolds shaped by brain–behavior dynamics; interventions reshape the geometry of these manifolds to stabilize healthy states. **B** Proposed closed-loop framework. N-of-1 clinical designs collect individualized data to train adaptive AI models that learn each patient’s manifold. These models guide and optimize personalized interventions in real time, dynamically aligning treatments to stabilize resilient trajectories.

Importantly, our proposed framework suggests a novel treatment goal: whereas conventional interventions typically aim to shift patients from one state to another, a manifold-based approach would instead target the geometry itself: re-engineering the underlying dynamical landscape to stabilize adaptive trajectories and prevent maladaptive ones. To formalize this engineering perspective, let the intrinsic fluctuations of noise-averaged state space be described by a system of ordinary differential equations:

1 $$\dot{x}=f(x)$$

where $x\in {R}^{n}$ denotes the system’s state (e.g., neural activity, cognitive load, features of psychopathology), $\dot{x}=\frac{{dx}}{{dt}}$ its temporal evolution, and $f(x)$ the intrinsic dynamics. An intervention can then be modeled as a control input $u$, yielding the general form

2 $$\dot{x}=f(x)+g(x,u)$$

where $g(x,u)$ captures how external perturbations (e.g., therapy, pharmacology, stimulation) modify the system’s trajectories. Such perturbations deform the latent manifold by altering the flow field defined by $f(x)$, thereby reshaping both the local stability and global accessibility of mental states. More concretely, when the system admits a gradient description:

3 $$\dot{x}=-\nabla V(x)-\nabla W(x,u)$$

where $V(x)$ represents the intrinsic energy landscape and $W(x,u)$ its modification under intervention, reshaping $V(x)$ alters the probability and stability of mental states, which mathematically defines our definition of manifold reshaping. This formulation complements network-theoretic models of mental disorders [5] by embedding feature interactions within a dynamical manifold framework. In the linear case where both $f$ and $g$ are linear, the system reduces to

4 $$\dot{x}={Ax}+{Bu}$$

where $A$ encodes the system structure, and $B$ defines how inputs influence dynamics. With optimal state feedback control $u=-{Kx}$, the closed-loop system becomes:

5 $$\dot{x}=(A-{BK})x$$

Here, the effective system matrix becomes ${A}_{{intervened}}=A-{BK}$, enabling stabilization of desired trajectories through adaptive intervention design.

To enable personalized psychiatry from this framework, we argue for a closed-loop paradigm in which interventions inform models, and models in turn guide subsequent interventions (Fig. 1B). The value of a model lies not only in its predictive accuracy but in its capacity to regulate and stabilize desired trajectories. According to the Good Regulator Theorem of Conant and Ashby [6], every effective regulator must embody a model of the system it regulates. Consequently, the focus of modeling attempts shifts from forecasting short-term mood fluctuations to constructing generative models that are sufficiently expressive to stabilize desirable attractors within the cognitive–affective manifold—such as remission, resilience, or relapse prevention. Beyond its theoretical appeal, this approach also provides a practically necessary framework for clinical application and may help address a central problem in neuroscience-based psychiatry: the lack of reproducible and prospective validation. In contrast to conventional approaches, closed-loop designs embed validation directly within the control process. Each adaptive intervention constitutes a prediction about how the system will respond to a defined perturbation, and the subsequent observation of that response provides immediate empirical feedback. This recursive prediction–perturbation–measurement cycle implements adaptive experimental design, ensuring that models are continuously tested, refined, and validated against real-world data.

Active interventions further provide a second key advantage: they dramatically improve sample efficiency: passive observation requires exponentially many samples to approximate the global landscape, $n\sim {e}^{\triangle V/T}$, where $n$ is the number of required samples, $\triangle V$ denotes the energy barrier separating attractor states, and $T$ is an effective noise or “temperature” parameter, following the Gibbs distribution $\pi (x)\propto {e}^{-\triangle V/T}$. In contrast, targeted perturbations can deliberately drive the system into undersampled regions, thereby reducing the sample complexity to $n\gtrsim {klog}(d)$, for a $k$-dimensional manifold embedded in ${R}^{d}$, where $k$ is the intrinsic dimensionality of the manifold and $d$ is the dimensionality of the ambient space. This formal relationship highlights the importance of interventional closed-loop designs in psychiatry.

Two theoretical and experimental aspects are central to this approach. First, hypotheses within this framework are formulated dynamically, in terms of the geometry and evolution of manifolds. They address properties such as manifold complexity, attractor depth, bifurcation thresholds, and hysteresis during recovery, and are tested through controlled perturbations that probe how the manifold evolves over time. Model validity is evaluated by whether the resulting trajectory changes—such as stabilization or transitions between attractors—align with model predictions and whether these geometric properties correspond to specific features of psychopathology. This approach differs fundamentally from traditional pre–post comparisons, which capture only static snapshots rather than the full trajectories of change. Second, the framework requires longitudinal, densely sampled data. Increasing evidence indicates that neuropsychiatric dynamics unfold on relatively fast timescales, and principles from signal processing show that sparse sampling introduces aliasing, preventing accurate reconstruction of underlying system dynamics [7].

Consequently, our approach prioritizes densely sampled, deeply phenotyped individuals monitored over extended periods (weeks to months) rather than large cross-sectional cohorts with limited temporal resolution. Analyses of thousands of daily ecological momentary assessment (EMA) items suggest that weekly sampling already satisfies Nyquist–Shannon criteria for most clinical purposes; more frequent measurement may be necessary in selected patients or phases, which can be determined adaptively [8]. AI tools can increasingly infer relevant manifold features from wearable passive data streams (e.g., heart-rate variability, activity, sleep), thereby reducing active patient burden to a minimum. When optimized, patient burden remains comparable to—or even lower than—current intensive outpatient or day-clinic programs. Because mobile apps, wearables, and cloud-based clinical platforms already exist at scale [9]; successful implementation therefore hinges primarily on establishing robust data-protection policies and clinical workflows rather than developing entirely new technological systems.

Third, successful implementation depends on the design of interventions. Controlling complex systems is rarely intuitive; it demands simulation and optimization [10]. Translation into clinical practice thus shifts the clinician’s role from symptom-oriented treatment selection toward training and deploying individualized “surrogate” AI models of each patient’s cognitive–affective manifold [11]. These surrogates can be initialized from foundational models pretrained on population-level data and fine-tuned using a short series of targeted N-of-1 perturbations (e.g., single-session therapy, single-dose pharmacological challenges, or brief neuromodulation combined with dense EMA and neuroimaging). AI-based controllers can model alternative perturbation scenarios, identify optimal interventions, and apply them across modalities such as neuromodulation, pharmacology, or psychotherapy [12].

We, therefore, propose a recursive framework consisting of: (i) designing N-of-1 longitudinal interventions; (ii) modeling individual brain–behavior dynamics; (iii) using generative AI to simulate perturbations and counterfactuals; and (iv) optimizing the next round of interventions based on model outputs. Within this loop, individualized therapies become a control engineering problem defined over latent manifolds inferred from real-time data.

This adaptive framework enables models to evolve into better regulators over time. Mental health is thus redefined not as the absence of features of psychopathology but as the ability to sustain adaptive trajectories under perturbation —that is, to recover and maintain resilience. The clinical aim thus shifts from feature reduction to stabilizing healthy dynamics within the cognitive–affective landscape, and psychopathology is reconceptualized as a loss of regulatory capacity, manifesting as rigid, maladaptive trajectories.

## Supplementary information

Supplementary Information
