# Supplementary material for: Re-engineering the disordered mind: clinical experimentation, dynamical systems, and AI for personalized psychiatry
Source: Neuropsychopharmacology. 2025 Dec 17;51(4):718–21. doi: 10.1038/s41386-025-02303-z (PMC12932708; doi:10.1038/s41386-025-02303-z)
Supplement: Supplementary file 1 — Supplementary Information [file 41386_2025_2303_MOESM1_ESM.docx]

**Supplementary Information**

**Supplementary Table 1.** Glossary of Core Concepts Across Computational, Clinical, and Dynamical Systems Perspectives

| **Concept** | **Computational Definition** | **Clinical Example** | **Clarification** |
| --- | --- | --- | --- |
| **Trajectory** | State trajectory *x(t)*; sequence in latent space describing system evolution. | Progression, relapse, or recovery of affective or cognitive-affective features. | The temporal evolution of a patient’s mental health. In computational terms, this represents a path through state space—a sequence of positions capturing the joint dynamics of neurocognitive and affective variables. |
| **Latent Space** | Internal state space representing hidden variables in the model. | Underlying cognitive–affective or neural states that are not directly observable. | A learned or inferred low-dimensional representation that encodes hidden mental processes. It provides a compact description of complex brain–behavior interactions. |
| **Manifold** | Low-dimensional surface constraining possible system trajectories. | The constrained “playing field” of cognitive-affective features e.g., a 3D surface capturing joint dynamics of arousal within the high dimensional neurobehavioral state space | The geometric structure that constrains possible trajectories of cognition and affect. Stable regions within this structure correspond to recurrent mental or emotional patterns. |
| **Energy Landscape** | Potential or cost function defining stability and transition barriers. | Barriers to or facilitators of therapeutic change. | A topographical metaphor in which attractors appear as valleys. Its geometry determines how easily a system can transition from maladaptive to adaptive states. |
| **Geometry** | Curvature, topology, and connectivity of the manifold. | Rigidity (sharp folds) vs. flexibility (smooth gradients) in emotional response. | The overall “shape” of the manifold. Smooth regions allow flexible transitions, while rugged or sharply curved regions limit transitions and promote persistence in maladaptive patterns. |
| **Attractor** | Fixed point, limit cycle, or stable trajectory toward which the trajectories converges. | Chronic low mood, anxiety loops, or resilient coping patterns. | A locally stable configuration of the system. Maladaptive attractors represent pathological dynamics, whereas adaptive attractors correspond to resilient and healthy functioning. |
